# Supplementary figures and images for: Identification of miRNAs and Their Targets in Cunninghamia lanceolata Under Low Phosphorus Stress Based on Small RNA and Degradome Sequencing
Source: Int J Mol Sci. 2025 Apr 12;26(8):3655. doi: 10.3390/ijms26083655 (PMC12027079; doi:10.3390/ijms26083655)

## SP\_result Conserved statistics

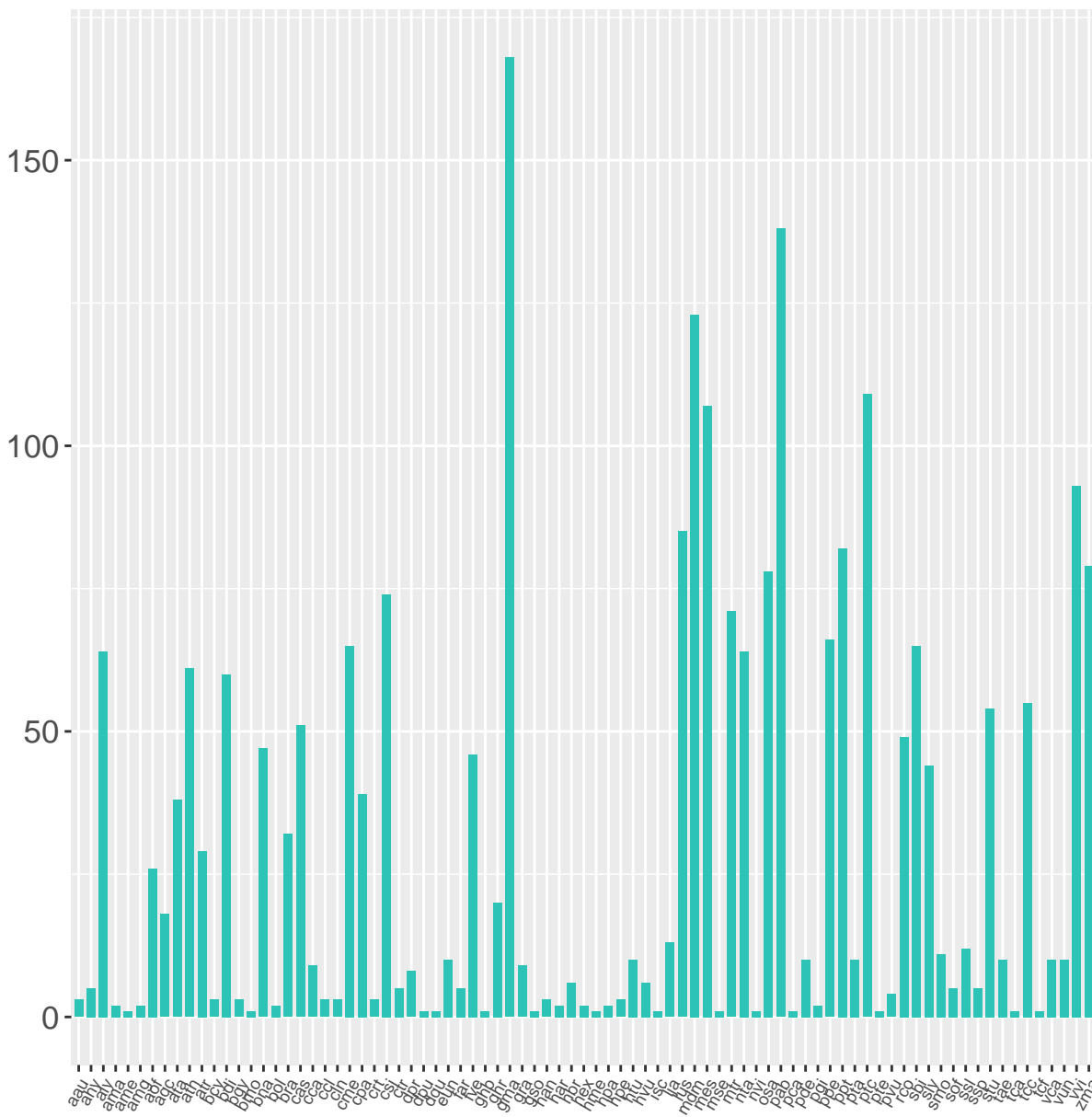

Supplement: Supplementary file 1 [file ijms-26-03655-s001.zip › Figure S1.pdf]

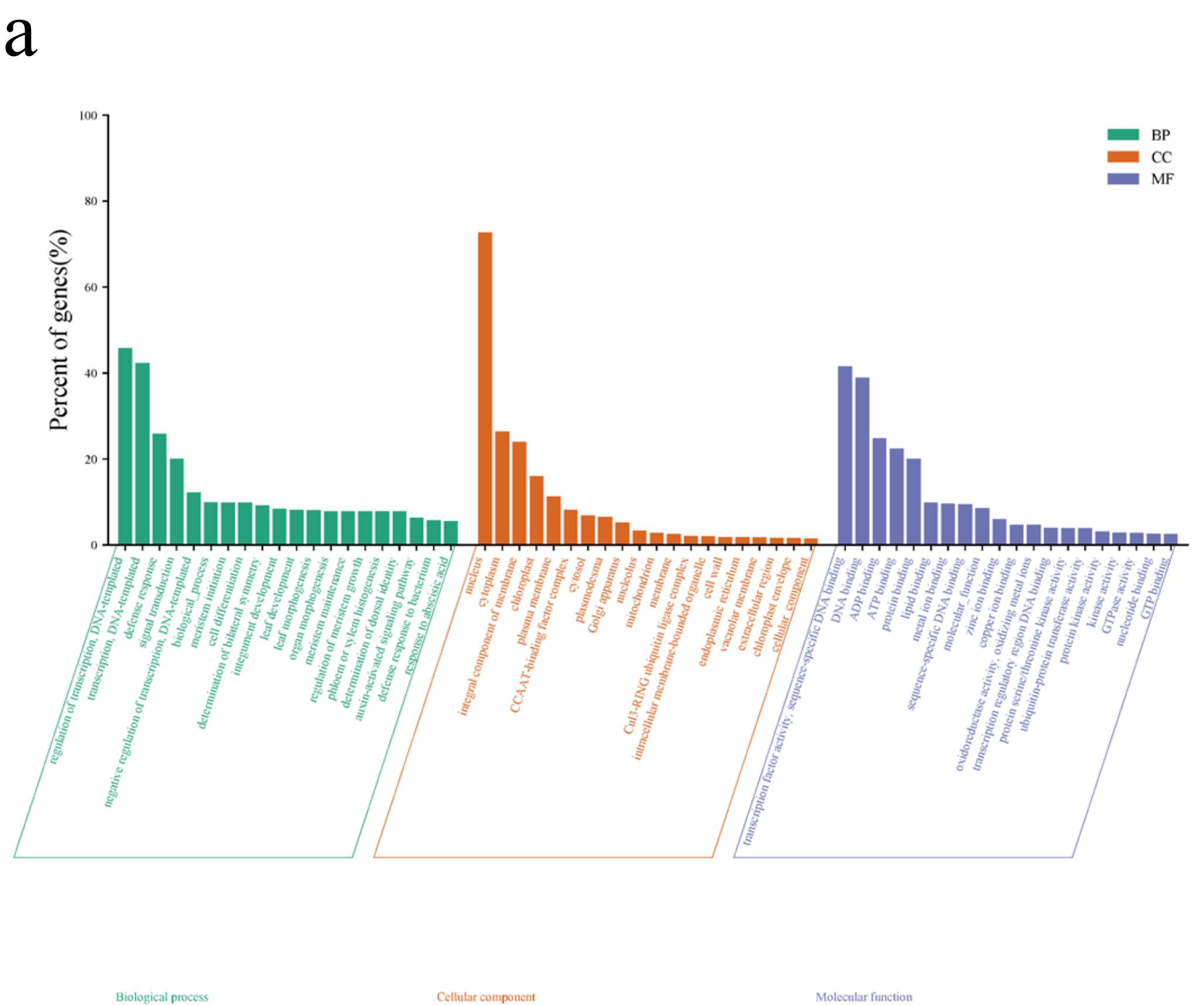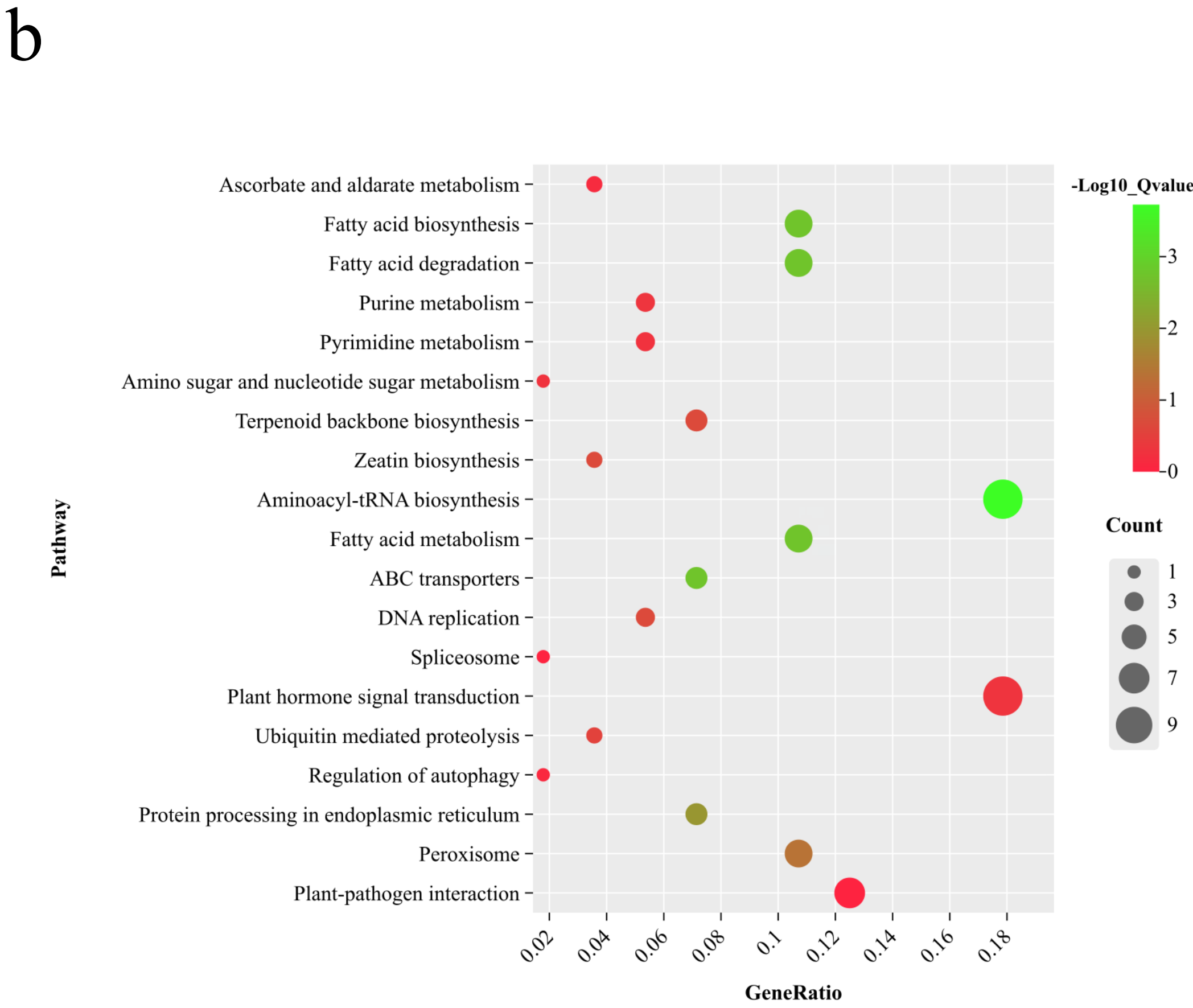

**Figure S2.** GO (a) and KEGG (b) enrichment analyses of DEM target genes in Chinese fir.

Supplement: Supplementary file 1 [file ijms-26-03655-s001.zip › Figure S2.pdf]

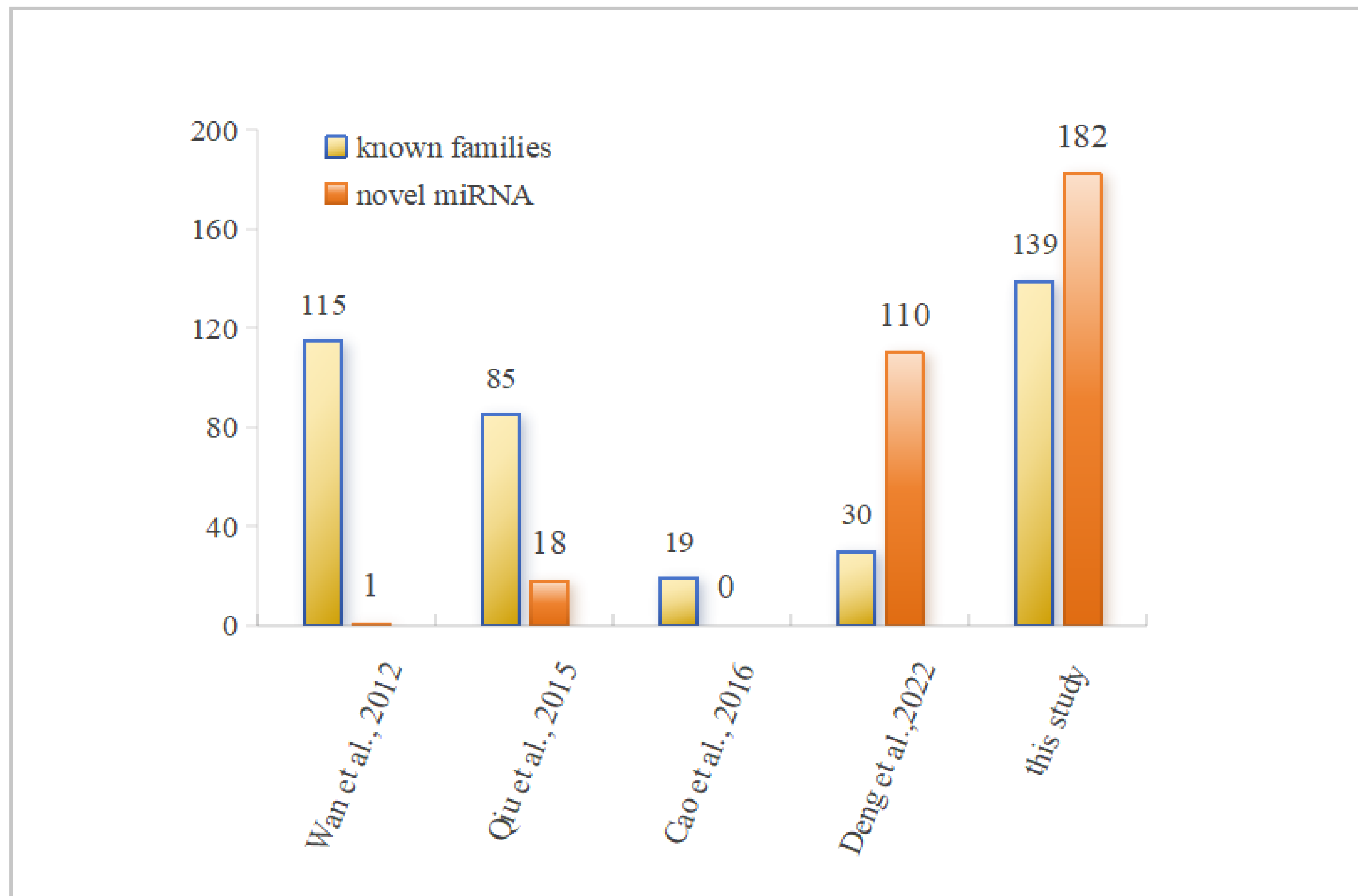

**Figure S3.** The distribution of miRNAs identified in different studies

Supplement: Supplementary file 1 [file ijms-26-03655-s001.zip › Figure S3.pdf]

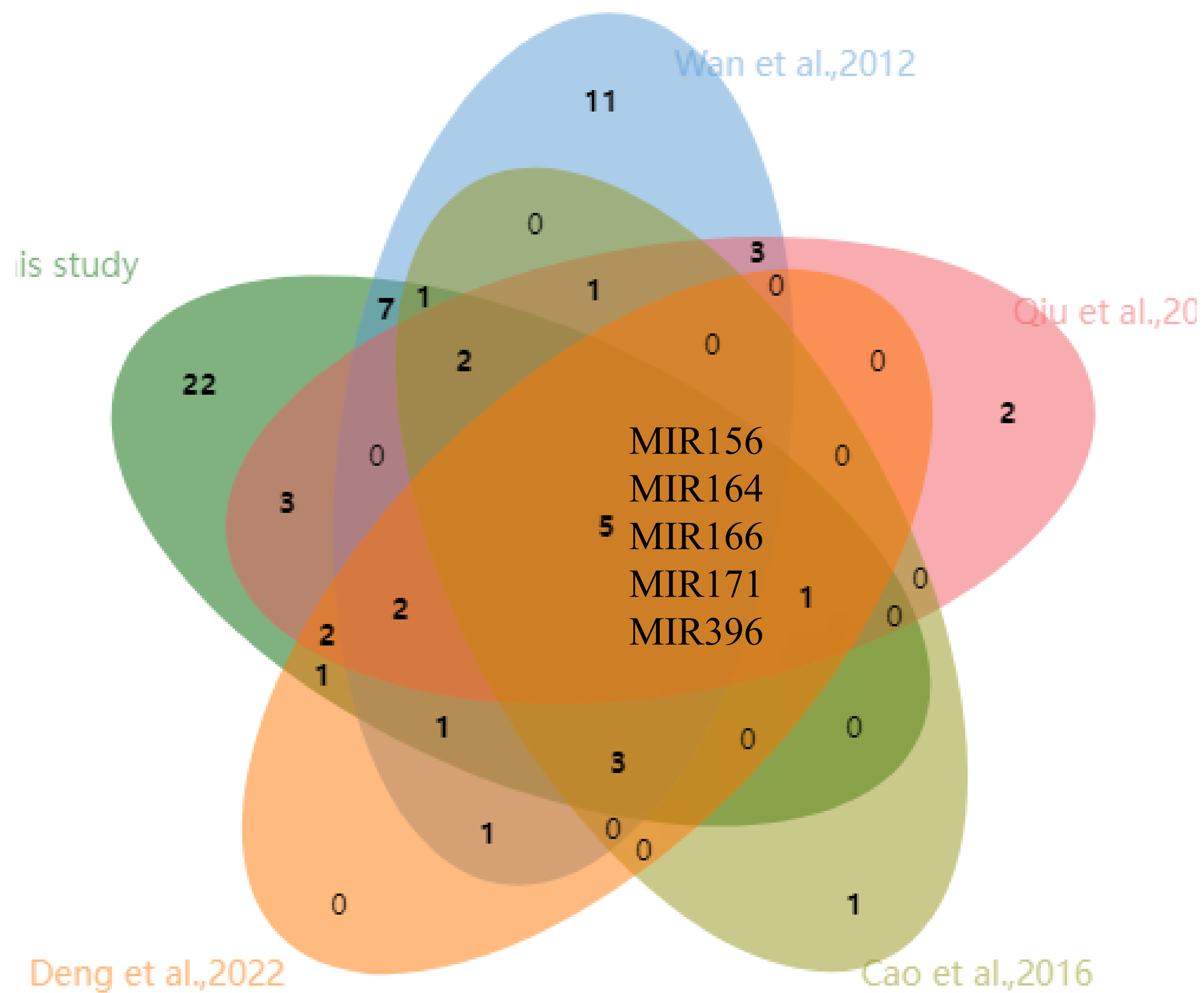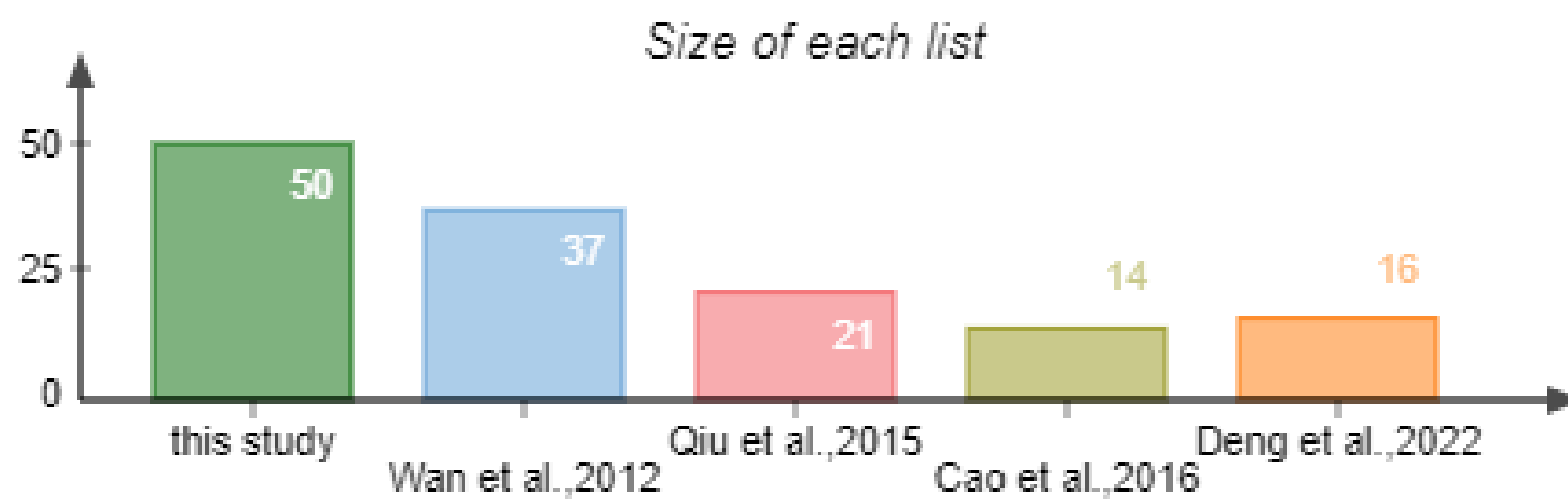

Supplement: Supplementary file 1 [file ijms-26-03655-s001.zip › Figure S4.pdf]
